# A CROSS-NATIONAL COMPARISON OF THE SOCIAL SUPPORT NETWORKS OF OLDER MEN AND WOMEN WHO LACK TRADITIONAL FAMILY TIES

**DOI:** 10.1093/geroni/igz038.2086

**Published:** 2019-11-08

**Authors:** Christine Mair, Kasey Knopp

**Affiliations:** University of Maryland, Baltimore County, Baltimore, Maryland, United States

## Abstract

Existing literature on “aging alone” focuses on potential lack of support to “kinless” older adults who do not have traditional family ties (e.g., child, spouse; Margolis & Verdery, 2018), as well as the ways in which childless or unpartnered older adults may construct non-kin networks of support (e.g., friendship; Djundeva et al., 2018; Mair, 2019). In addition, older men’s and women’s social networks vary, with women reporting more network growth than men and potentially lower family involvement (Schwartz & Litwin, 2018). Finally, patterns of support (e.g., family care, friend interactions) differ by country context. However, it is unknown if and how the social networks of older adults who lack traditional family ties may differ by gender, as well as what forms of cross-national variation exist in these patterns. Using data from the Survey of Health, Ageing and Retirement in Europe (SHARE, N=17 nations, N=53,247 adults aged 50+), we take advantage of a unique social support network module in this cross-national dataset to compare closeness, proximity, quality, and type of ties by gender among older childless and unpartnered men and women by country. Among those without traditional family ties, we find that older women may be advantaged in terms of social support compared to older men, but that this advantage varies by nation. We discuss the details and implications of these results regarding potential policy implications about the differential risks faced by older men and women who lack traditional family ties in various country contexts.

